# Development of an observational instrument to determine variations in the patient care process and patient flow among emergency physicians and internists at the emergency department

**DOI:** 10.1186/1865-1380-6-1

**Published:** 2013-01-15

**Authors:** Daisy Roxanna Johanna Christina Koks, Maartje Elisabeth Zonderland, Christian Heringhaus

**Affiliations:** 1 Emergency Department, Leiden University Medical Center, Albinusdreef 2, Postbox 9600, 2300 RC Leiden, The Netherlands; 2 Stochastic Operations Research & Center for Healthcare Operations Improvement and Research, University of Twente, Postbox 217, 7500, AE, Enschede, The Netherlands

**Keywords:** Emergency department, Patient flow, Patient care process, Length of stay, Emergency physicians, Internists

## Abstract

**Background:**

The increasing demand for acute care and restructuring of hospitals resulting in emergency department (ED) closures and fewer inpatient beds are reasons to improve ED efficiency. The approach towards the patient care process varies among doctors. The objective of this study was to determine variations in the patient care process and patient flow among emergency physicians (EP’s) and internists at the ED of Leiden University Medical Centre (LUMC), the Netherlands.

**Methods:**

An observational instrument was developed during a pilot study at the LUMC ED, following observations of activities performed by EP’s and internists. The instrument divides all different types of activities a clinician can perform on the ED into eight categories. Using the observational instrument, their activities were observed and registered for 10 separate days. Primary outcomes were defined as the time spend on the eight separate activity categories, the total length of stay (LOS) and the number of patients seen during an interval. Secondary outcomes were general observations of working routine features that determine patient flow at the ED. The obtained data were analyzed into SPSS.

**Results:**

Ten doctors were observed during a total of ± 36 hours in which 42 patients were seen. Although EP’s were observed for a shorter period of time than internists (13:48 vs. 22:10 hrs, -38%), they saw more patients (26 vs. 16, +62%). EP’s tended to spend a higher proportion of their time on patient contact than internists (27.2% vs. 17.3%, p = 0.06). Both groups dedicated the highest proportion of their time to documentation (31.5% and 33.4%, p = 0.75) and had little communication with ED nurses (3.7% and 2.4% p = 0.57). The average LOS of internal patients was higher than that of EP’s patients (5.25 ± sd 1:33 and 2.26 ± sd 1:32 hours). Internists occupied more treatment rooms at the same time (2.41 vs. 2.08, p < 0.00) and followed a more sequential working routine.

**Conclusions:**

This paper describes the determination of variations in the ED care process and patient flow among EP’s and internists by an observational instrument. A pilot study with the instrument showed variations in the patient care process and patient flow among the two groups at the LUMC ED.

## Background

Emergency department (ED) overcrowding in Western countries is an ongoing challenge and has several adverse outcomes associated with it, such as increased mortality rates [1-3]. The increasing demand for acute care [4] and the restructuring of hospitals resulting in ED closures and fewer inpatient beds [5] are immediate reasons to improve ED efficiency, so that the current situation will not get worse and hopefully improves. The approach towards patient care and the sequence of steps in the care process varies among doctors and is dependent of the health care setting in which they provide care [6], such as the ED, inpatient wards, public health clinic and hospital outpatient clinic (Table 1).

**Table 1:** **Comparison of ED and inpatient care settings**[6]

**Emergency department**	**Inpatients wards**
Low moderate and high urgency	Low and moderate urgency
Undifferentiated patient	Admitted Patients
Approach directed on complaint	Approach directed on preliminary diagnosis
Diagnostic tests ordered are of moderate or high urgency	Diagnostic tests ordered are of low, moderate or high urgency
Results of diagnostic tests available within minutes to hours	Results of diagnostic tests available within hours to days
Patient evaluations of several patients	Patient evaluations are scheduled
Parallel evaluations of several patients	Evalauation of one patient at a time

**Table 2:** Observational instrument: main categories and related activities

**Main category**	**Activities**
Patient contact	Face to face patient contact: history and physical examination, explanation of diagnosic and treatment
Documentation	Writing medical status
	Writing letter
	Reclaiming medical history
	Financial settlement
	Ordering medical tests and reviewing results
	Arranging admission
	Arranging discharge
Consult supervisor	Consultation of supervisor
	Transmission of patient to doctor from own specialty
	Contacting supervisor or doctor from own specialties
Consult others	Consult colleagues
	Transmission of patient to doctor from other specialty
	Contacting colleagues or doctors from other specialties
Communication with nurse	Deliberating with ED nurse
Waiting	Waiting for test result availability or other ED staff to finish
Absence from ED	Absence from ED to perform duties on other hospital locations, such as the inpatient wards and outpatient clinics or because of scheduled education moments
Other (specify)	Other, not specified in the activities above, such as taking a break

**Table 3:** Differences between patient presentation by triage category between emergency physicians and internists

**Emergency physicians**		**Internists**		
**Triage category **[7]	**Patient presentation percentage (n)**	**Patient presentation percentage (n)**	**Odds Ratio**	**P-value**
Green	38% (10)	13% (2)	4.38	0.07
Yellow	35% (9)	56% (9)	0.41	017
Orange	23% (6)	31% (5)	0.66	0.43
Red	4% (1)	0% (0)	n/a	0.43
Total	100% (26)	100% (16)		

**Table 4:** Time investment of emergency physicians and internists per main category

**Emergency physicians**			**Internists**		
**Main category**	**Total (min.)**	Mean percentage* (sd)	**Total (min.)**	Mean percentage* (sd)	**P-value (95% BI)**
Patient contact	231	27.2% (7.5%)	229	17.3% (6.5%)	0.06 (-0.41; 20.09)
Documentation	252	31.5% (9.2%)	449	33.4% (9.1%)	0.75 (-15.16; 11.44)
Consult supervisor	124	14.4% (5.4%)	129	9.5% (4.1%)	0.15 (-2,10; 11.82)
Consult others	43	5.3% (4.6%)	139	9.9% (4.8%)	0.16 (-11.45; 2.17)
Communicating nurse	30	3.7% (4.5%)	31	2.4% (2.0%)	0.57 (-3.70; 6.38)
Waiting	28	2.8% (4.6%)	116	9.7% (13.4%)	0.33 (-23.31; 9.47)
Absence from ED	0	0.0% (0.0%)	101	7.9% (13.7%)	0.26 (-24.93; 9.05)
Other	120	15.1% (11.8%)	136	9.7% (5.7%)	0.39 (-8.19; 18.87)
Total	828	100%	1330	100%	n/a
Not registered	46		19		

*Is the mean of the percentages of all days ≠ the percentage of the total.

**Table 5:** Length of stay differences between patients treated by emergency physicians or internists

**Emergency physicians**			**Internists**		
**Length of stay**			**Length of stay**		
**Triage category**	**Mean (hours)**	**sd (hours)**	**Mean (hours)**	**sd (hours)**	**P-value**
Green	1:55	1:11	4:16	2:32	0.05
Yellow	2:14	1:14	5:49	0:58	<0.00
Orange	3:17	2:13	5:10	2:04	0.18
Red*	4:24	n/a	n/a	n/a	n/a
Total	2:26	1:33	5:25	1:33	<0.00

*Because only one patient was triaged in the red category, no sd or p-value could be calculated.

Since emergency physicians (EP’s) work only in the ED while other specialists spend most of their time in different care settings, it is likely that the working routines of EP’s are more adjusted to the ED setting. Furthermore, EP’s are trained to treat a broad spectrum of patients and their complaints, while other specialists focus on a specific domain or part of the body and are trained to treat either surgical or medical patients. The objective of this study was to determine variations in approaches towards the urgent patient care process among EP’s and internists by an observational instrument. A small pilot study with the instrument was performed. The outcomes will be related to factors influencing ED patient flow.

## Methods

### Study design and setting

The observational instrument (Table 2) was developed after numerous observations of activities performed by clinicians working at the Leiden University Medical Centre (LUMC) and extensively discussed with ED staff. The instrument divides all different types of activities a doctor can perform at an ED into 8 categories. The pilot study with the instrument was also carried out at the LUMC ED.

The LUMC is a Dutch tertiary care university hospital with ±30,000 annual ED visits and an admission rate of 25%. Patients are triaged by a qualified nurse using the Manchester triage system [7]. In the United States, Australia and the United Kingdom ED’s are staffed EP’s while medical specialists only attend the ED if consulted. Since the education of dedicated EP’s started only a couple of years ago in the Netherlands, it is not yet possible to find enough employees for continuous ED staffing. The LUMC is in a transition phase with an increasing number of EP’s taking over workload from other specialties. Therefore only a part of the ED shifts (day and evening during weekdays) at the LUMC is covered by EP’s. The majority of patients at the ED who are not treated by the EP’s are seen by internists.

### Data collection and analysis

In the pilot study both groups were observed by an independent observer 10 days (5 days each) in the period January – March 2010. Every minute, the doctor’s activity was registered by the observer, together with the patient’s ID. When a doctor was doing several things during a minute, the activity which took the longest time was recorded. The length of the period in which the observations were carried out varied from 2 to 6 hours, due to the (in-)availability of the observers. The observers followed one doctor at a time, who was blinded with regard to the purpose of the study and was instructed to carry out his normal work routine. During and after the observations, the doctors did not receive any feedback. The doctors, male and female, were selected randomly and had 2 to 6 years of experience working as a resident. All doctors selected agreed to cooperate. After the observations, all activities recorded were divided in the 8 categories of the observational instrument. For each patient included in the study the length of stay (LOS) and triage category were acquired via the ED administrative and clinical data system. The number of patients seen by the doctor during the observation period was also recorded. Additionally, general observations of working routine features were made. The obtained data were analyzed into SPSS version 16.0 (SPSS Inc., Chicago, IL). Statistical analyses were conducted using independent samples t-tests, two-way chi-square and logistic binary regression analyses.

## Results

The observations took a total of *±*36 hours (mean 3:42, sd 1:11 hours) in which 42 patients were seen. Although EP’s were observed for a shorter period of time than internists (13:48 vs. 22:10 hours, -38%), they saw more patients (26 vs. 16, +62%). Due to short periods of absence of the observers (bathroom visits etc.) 65 minutes were not recorded.

### Triage categories

A significant difference in the distribution of the triage categories between both groups was not found (p = 0.305). However, when comparing both groups per triage category, EP’s relatively tended to treat more patients from the green triage category than internists, although this difference was not significant (p = 0.07) (Table 3).

### Distribution over categories of the observational instrument

In Table 4 the total time recorded per category of the observational instrument is given for both groups of doctors. EP’s tended to spend a higher proportion of their time on face to face patient contact than internists (27.2% vs. 17.3%, not significant: p = 0.06). EP’s did not leave the ED during observations, while internists left the ED in 7.9% of the observed time, mainly since they were also responsible for patients in other hospital departments, were required to attend education sessions, or had to attend meetings where (their) patients were discussed. Internists spended a smaller proportion of their time to consult their supervisor (9.5% vs. 14.4%, p = 0.15). Both groups spend the highest proportion of their time on documentation (31.5% and 33.4%, p = 0.75) and had minimal communication with the ED nurses (3.7% and 2.4% p = 0.57). Waiting during the observations was in both groups mostly related to diagnostic tests that were not yet performed or reviewed.

### LOS and the number of treatment rooms occupied

The LOS for patients treated by internists was significantly higher (5:25 vs 2:26 hrs, p *<* 0.00) (Table 5). The number of treatment rooms simultaneously occupied by one doctor was significantly higher for internists (2.41 vs. 2.08, p *<* 0.00).

### General observations

Variations in the sequence of steps taken in the patient assessment were perceived. Internists generally followed the traditional approach of assessing history, performing physical and diagnostic tests, determining diagnosis and initiating treatment. EP’s started more often (53% vs. 33% of their patients, p = 0.418) with physical examination (including performing the structured ABCDE approach [8] and vital signs if necessary), followed by immediate ordering diagnostic tests and initiating treatment, prior to obtaining a detailed history.

Other observations were that the care process of patients who were subsequently admitted at an inpatient ward was often completed at the ED, although the urgency indication was no longer present. Because of the little communication between doctors and ED nurses, the next steps in the care process were not transparent and both doctors and nurses were waiting for each other to proceed. EP’s saw patients and received direct supervision. In contrast internists discussed most of their patients by phone with their supervisor and ‘collected’ several patients before they consulted their supervisors, so several patients could be discussed at once. Not only the difference in the order of the sequence of steps taken in the patient care process was an outcome of the pilot study, it was also observed that the internists followed a more sequential working routine by ordering diagnostic tests. For instance, most EP’s would order several diagnostic tests parallel and at once, while the internists would order one test at a time and, depending on the test results, would then decide on the next test to order.

## Discussion

The objective of this study was to determine variations in the patient care process among EP’s and internists through a pilot study with the observational instrument. Even though a small number of patients was included in the pilot study, many results were significant and showed the instrument’s potential. For some patients included the care process had started before the beginning of the observation, or ended after the observation terminated. This could have confounded the results. A limitation of the pilot study was that the complexity of the illness of the patients was not recorded, a factor which could have influenced the patient’s LOS to a great extent. It was difficult to account for the overall crowding of the hospital, even though this could also have influenced the patient’s LOS at the ED.

Several other factors can be deduced from this study that likely influenced the patients LOS at the ED. Internists tended to ‘collect’ patients in order to discuss them with their supervisor all at once, even though the care process may be well advanced at that point. If the supervisor suggests a different treatment, a lot of time is wasted and unnecessary work done, which is patient unfriendly as well. The decision to admit the patient is usually taken during this discussion, while this is often evident in an earlier stage of the care process. As a consequence, the time consuming procedure of finding an inpatient bed is started at a later point in time. Furthermore, an increase in communication between the doctors and ED nurses could improve the patient’s LOS, just as a decrease in the amount of administrative activities the doctors are required to perform.

**Figure 1:**
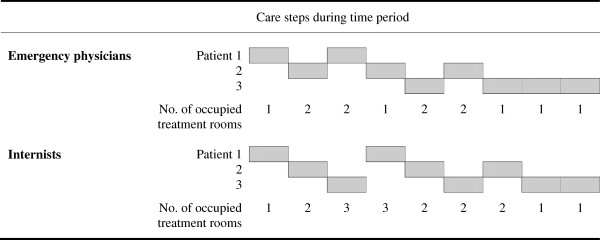
**Influence of sequential and parallel care processes on the number of occupied treatment rooms.** Legend: We have 2 doctors both treating 3 patients, who respectively require 2, 3 and 4 care steps of 1 time unit. Both doctors need the same time to complete all 3 care processes (9 time units), but the doctor who uses patient rooms more sequentially occupies less treatment rooms at the same time (on average 1.44 vs. 1.89). With a more sequential use of treatment rooms we refer to a treatment process where the doctor completes as many activities as possible at once by one patient, then in the time he has to wait (for instance for test results) starts treating another patient, and then as soon as possible finishes the treatment process of the first patient. Even in this small example we see the effect on ED patient flow. The mean patient LOS for the first doctor equals 4.33 time units, and the mean waiting time is 1.33 time units. For the second doctor, the mean LOS is 5.67 time units and the mean waiting time equals 2.67 time units. The broad education of emergency physicians allows them to select patients from a larger group than the internists, and follow a work routine which is more like the first doctor.

Since internists kept more treatment rooms occupied for a longer period of time, less treatment rooms were available for other doctors to receive new patients. This in turn results in longer waiting times for patients who are still in the waiting room. Consider the example in Figure 1.

In a follow-up study the observation period will be extended, observation lengths will be more evenly distributed, and a severity of illness classification system will be included. We also aim to incorporate the overall crowding of the hospital and quantify its effect on the patient’s LOS. This hopefully enables us to meticulously observe the different approaches of physicians towards the urgent patient’s care process and their consequences for ED patient flow. Our ultimate goal is to define uniform ED treatment protocols from these results and maximize the ED efficiency.

## Conclusions

This paper describes the determination of variations in the urgent patient care process among EP’s and internists at the ED through a small pilot study with the observational instrument. Internists ordered diagnostic tests more sequentially than EP’s and used more patient rooms at the same time. Also, their patients had a higher LOS. In order to decrease the LOS and reduce overcrowding at the LUMC ED, communication between doctors and nurses should be stimulated, just as an earlier consultation between residents of internal medicine and their supervisors. In order to keep treatment rooms available for urgent patients the continuation of care of discharged (ex-ED) patients at another department, instead of in the ED, should be encouraged. Further research to define uniform ED treatment protocols could be valuable. Meanwhile, even based on this small pilot study, the first steps in the LUMC have been taken to improve the patient flow at the ED by, for example, introducing a hospital-wide patient data management system, adjusting the supervision model of internists and introducing the ABCDE approach for other ED clinicians.

## Abbreviations

ED: Emergency Department; LUMC: Leiden University Medical Centre; LOS: Length Of Stay; EP: Emergency physician.

## Competing interests

The authors declare that they have no financial or non-financial competing interests.

## Authors’ contributions

DK developed the study design and setting, acquired the data, performed the statistical analyses and drafted the manuscript. MZ developed the study design and setting, acquired the data, performed the statistical analyses, interpreted the data, drafted the manuscript, and revised the manuscript critically. CH developed the study design and setting, interpreted the data and revised the manuscript critically. All authors read and approved the final manuscript.

## References

[B1] Guttmann A Schull MJ Vermeulen MJ Stukel TA Association between waiting times and short term mortality and hospital admission after departure from emergency department: population based cohort study from Ontario, Canada Br Med J 2011 342 d2983 10.1136/bmj.d2983 21632665 PMC3106148

[B2] Hoot NR Aronsky D Systematic review of emergency department crowding: causes, effects, and solutions Ann Emerg Med 2008 52 126 136 10.1016/j.annemergmed.2008.03.014 18433933 PMC7340358

[B3] Huang Q Thind A Dreyer JF Zaric GS The impact of delays to admission from the emergency department on inpatient outcomes BioMed Central Emergency Medicine 2010 10 16 20618934 10.1186/1471-227X-10-16 PMC2912828

[B4] Moskop JC Sklar DP Geiderman JM Schears RM Bookman KJ Emergency department crowding, part 1 - concept, causes, and moral consequences Ann Emerg Med 2009 53 605 611 10.1016/j.annemergmed.2008.09.019 19027193

[B5] Schull MJ Szalai J Schwartz B Redelmeier DA Emergency department overcrowding following systematic hospital restructuring: trends at twenty hospitals of ten years Acad Emerg Med 2001 8 1037 1043 10.1111/j.1553-2712.2001.tb01112.x 11691665

[B6] Clerkship Directors in Emergency Medicine Wald DA Differences between the emergency department, the office, and the inpatient setting Emergency medicine clerkship primer, a manual for medical students 2008 Lansing 13 16

[B7] Van der Wulp I van Baar ME Schrijvers AJP Reliability and validity of the Manchester Triage System in a general emergency department patient population in the Netherlands: results of a simulation study Emerg Med J 2008 25 431 434 10.1136/emj.2007.055228 18573959

[B8] Committee on Trauma, American College of Surgeons ATLS: advanced trauma life support program for doctors 2008 8 Chicago

